# “Do It Yourself” Microbial Cultivation Techniques for Synthetic and Systems Biology: Cheap, Fun, and Flexible

**DOI:** 10.3389/fmicb.2018.01666

**Published:** 2018-07-30

**Authors:** Teuta Pilizota, Ya-Tang Yang

**Affiliations:** ^1^Centre for Synthetic and Systems Biology, School of Biological Sciences, University of Edinburgh, Edinburgh, United Kingdom; ^2^Department of Electrical Engineering, National Tsing Hua University, Hsinchu, Taiwan

**Keywords:** bioreactor, synthetic biology, systems biology, microbial cultivation, evolution, optogenetics

## Abstract

With the emergence of inexpensive 3D printing technology, open-source platforms for electronic prototyping and single-board computers, “Do it Yourself” (DIY) approaches to the cultivation of microbial cultures are becoming more feasible, user-friendly, and thus wider spread. In this perspective, we survey some of these approaches, as well as add-on solutions to commercial instruments for synthetic and system biology applications. We discuss different cultivation designs, including capabilities and limitations. Our intention is to encourage the reader to consider the DIY solutions. Overall, custom cultivation devices offer controlled growth environments with in-line monitoring of, for example, optical density, fluorescence, pH, and dissolved oxygen, all at affordable prices. Moreover, they offer a great degree of flexibility for different applications and requirements and are fun to design and construct. We include several illustrative examples, such as gaining optogenetic control and adaptive laboratory evolution experiments.

## Introduction

Arguably, microbiology is experiencing a renaissance driven by several applied and basic research fields, e.g., combating antimicrobial resistance and using microbes in industrial biotechnology relies on successes of systems and synthetic biology that strive to understand and engineer microorganisms. At the basis of these research efforts is the ability to culture microorganisms with sufficient flexibility and throughput. For example, synthetic biology is recognized as an emerging technology able to combine research excellence with the power of businesses to develop novel products to drive economic growth (Pleiss, 2006). The vision rests on the ability to offer enhanced control of gene expression and metabolic pathways, either through modulating existing pathways or by introducing novel pathways in a given organism (Pleiss, 2006). Thus, the engineered microorganisms can effectively become factories, sustainably producing a range of products, for example, biologics and diagnostics (Scognamiglio et al., 2015). The process of strain engineering passes through a design–build–test–learn iterative cycle, which, at the start, is high throughput but involves working with small culture volumes that can later be scaled up for commercial and industrial purposes. Similarly, to decipher complex interactions of biological systems and identify mathematical models that best depict their behavior, systems biology benefits from large datasets, which are often captured working with small culture volumes that require experiment-specific control of growth environments. Thus, small volumes at the start that can lateer be scaled up, very specific environmental control of growing cultures, and an increasing range of microbes that are of great interest, make it almost impossible to purchase a microbial cultivation unit that will “do it all." However, inexpensive 3D printing technology and modular open-source platforms for electronic prototyping offer affordable and straightforward building blocks that can expand how we culture microbes, either by offering inexpensive alternatives to commercial products or novel, commercially unavailable, solutions. We survey some of the examples with the intention of encouraging the reader to consider “Do-It-Yourself" (DIY) approaches. We focus on small-volume cultivation (ranging from 100 μl to sub-liter scale), simply because it is frequently used in synthetic and systems microbiology. However, DIY approaches are applicable for medium to large culture volumes as well. We also note that microfluidic-based cultivation technology has the potential to further reduce culture volumes and increase throughput, and it is increasingly developed (Ferry et al., 2011; Han et al., 2013; Hegab et al., 2013; Yang and Wang, 2016).

## Small-Volume Cultivation Design Considerations

Arguably, two most frequent small-volume microbial cultivation units are a bioreactor and a plate-reader. A bioreactor is a vessel that allows controlled growth of a microbial culture, for example, through the supply of fresh media and fixed environmental oxygen levels. Typical bioreactors come with a stirring unit to mix the cells in the culture and achieve full oxygenation of the medium, as well as with in-line monitoring capabilities, e.g., turbidity measurements. Bioreactors are operated in the so-called “fed-batch” or “chemostat” mode (Novick and Szilard, 1950). In the fed-batch mode, the cells are given a fixed amount of nutrients and allowed to grow until nutrients are depleted. In the chemostat mode, fresh medium is constantly supplied while the cells are removed at a constant rate so that a biomass steady state is reached. The microplate or microtiter plate readers are also widely used for gene expression measurement and strain and medium optimization (Zaslaver et al., 2009). Microplates contain a standard number of wells (24, 48, 96, or 384); typically, each well contains few 100 μl.

**FIGURE 1:**
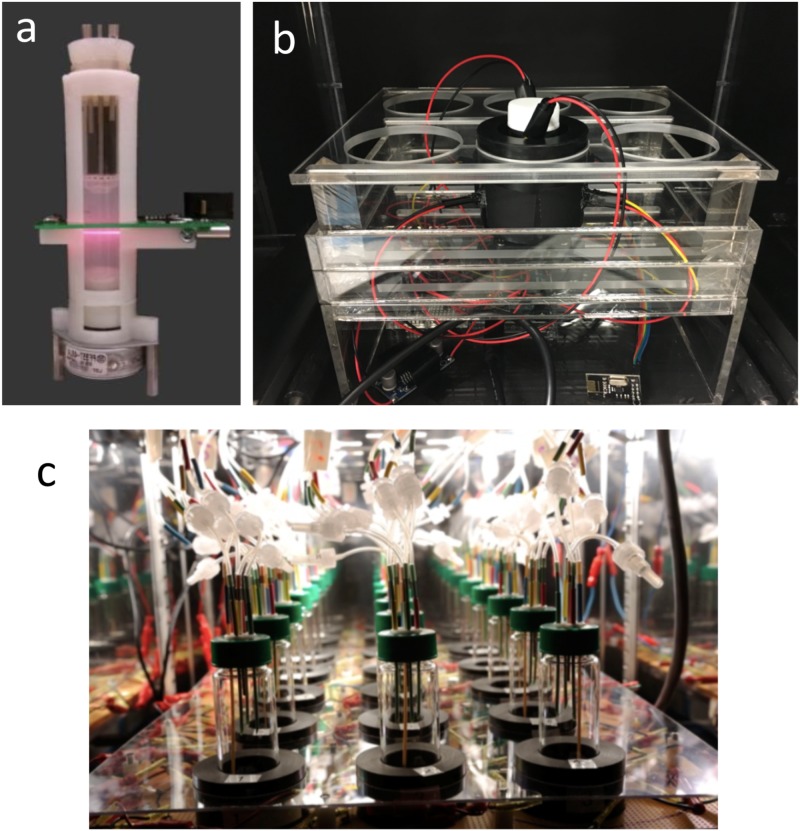
Custom bioreactors for synthetic and system biology. **(a)** “Turbidostat” built from the 3D printed holder with a laser diode and photodetector installed for optical density measurement (Takahashi et al., 2014). The culture vial is connected to a 3D printed syringe pump to enact the turbidostat mode. American Society of Chemistry. Reproduced with permission. **(b)** Optogenetic mini photo bioreactor (Wang and Yang, 2017). The device integrates light source for control of gene expression and optical density and fluorescence detection for monitoring microbial growth and gene expression. American Society of Chemistry. Reproduced with permission. **(c)** Morbidostat built for adaptive laboratory evolution (Toprak et al., 2012). The device integrates optical density measurements for monitoring bacterial cell concentration and implements a feedback algorithm to adjust the drug concentration delivered via the tubing connected to the vessel from the top (**Figure 3C** gives the feedback scheme). Nature Publishing Group. Reproduced with permission.

**FIGURE 2:**
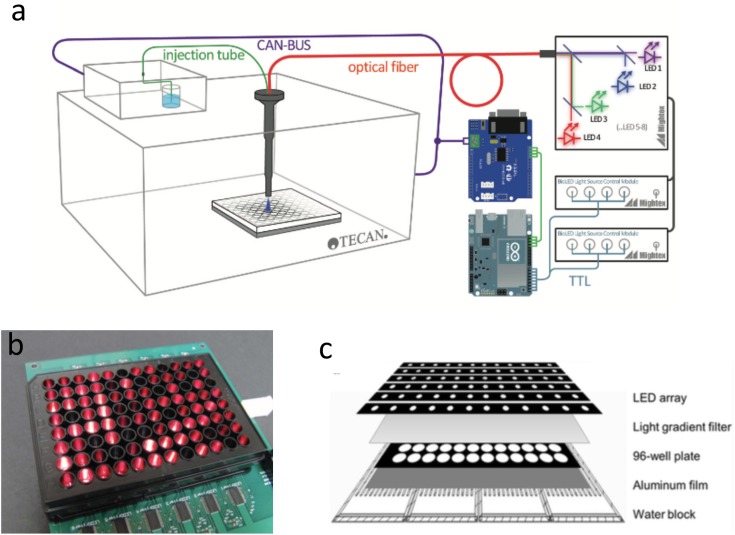
Microplate photobioreactors. **(a)** Add-on illumination path for a Tecan plate reader (Richter et al., 2015). Royal Society of Chemistry. Reproduced with permission. **(b)** An assembled optical microplate for microalgae cultivation (Chen et al., 2012). Royal Society of Chemistry. Reproduced with permission. **(c)** Layered assembly for the “Photobiobox,” a high-throughput solution for microalgal screening and culture optimization (Heo et al., 2015). Elsevier Ltd. Reproduced with permission.

Both bioreactors and microplate readers are commercially available, but as the cost of DIY electronic components and 3D printing technology progressively decreases, development of DIY bioreactors, microplate readers, and add-ons to commercial instruments is becoming feasible. Arguably, problems that can benefit most from DIY culturing technology require a great degree of flexibility or levels of control that are not readily available commercially. To give the reader ballpark numbers, 3D printers now come at $1500 USD and plastic consumables are on the order of 2D printing consumables. Arduino and Raspberry Pi microcontrollers range from $10 to $50. Lego Mindstorms Robots cost ∼$300 and come with a controller box and some Lego parts included. To give a rough estimate of total cost, a previously reported optogenetic bioreactor came under $300 (Wang and Yang, 2017), and an adaptive laboratory evolution robot at roughly $2500 (Arias-Castro, 2017). Below, we discuss examples of custom build bioreactors (Toprak et al., 2013; Takahashi et al., 2014; Wang and Yang, 2017) (**Figures 1a–c**) and microplate readers (Chen et al., 2012; Heo et al., 2015; Richter et al., 2015) (**Figures 2a–c**), all of which needed to satisfy few common requirements.

### Measuring Cell Number

There are several typical challenges that most reported examples of DIY bioreactors and microplate readers need to overcome or take into account (Klöckner and Büchs, 2012). First is the implementation of turbidity measurements of a microbial culture, i.e., optical density (OD) measurements. Measuring OD is the most standard way of assessing the growth of microbial cells in a culture, and OD is assumed to be proportional to the number of cells in the culture.

The OD density measurement is traditionally done with a light source and a photodetector. The assumption of linearity holds true in the so-called “single-scattering” regime, i.e., when the number of microbial cells in the culture is sufficiently low for the light to scatter off each cell only once as it passes through the sample. At higher cell culture densities, the so-called “multiple scattering” regime (usually above 10^8^ ml^−1^ cell concentration), OD measurements are no longer a good indication of the cell number and alternatives methods, such as direct counting or the so-called cell growth quantifier, should be used (Koch, 1970; Bruder et al., 2016; Stevenson et al., 2016). The limitation of the OD measurements is particularly relevant for fed-batch bioreactors, microplate readers, or applications where cell size changes, and changes in the index of refraction of the medium or cells are expected (Stevenson et al., 2016). The recently reported cell growth quantifier offers the possibility of extending the cell concentration measurements into the multiple-scattering regime by detecting the backscattered light rather than the transmitted (Schmidt-Hager et al., 2014; Bruder et al., 2016). Similar can be achieved by detecting *Escherichia coli*’s auto-fluorescence, linked with the secretion of flavins (Mihalcescu et al., 2015). Flavins are auto-fluorescent in the green region of the visible spectrum (peaking at ∼530 nm) and the time derivative of the auto-fluorescence scales linearly with a bacterial concentration in the ∼6⋅10^6^ to 10^9^ ml^−1^ range. Thus, when not working with cells expressing green fluorescent proteins, monitoring flavin auto-fluorescence extend the concentration ranges of OD measurements (Mihalcescu et al., 2015). On the other end of the scale, microbial cultures of very low densities will scatter very little light and the detection is limited by signal-to-noise ratio. An alternative approach to estimating cell number in low-density microbial cultures is based on bioluminescence photon counting (Kishony and Leibler, 2003). Naturally occurring luminescent bacteria are rare, but the biochemistry and genetics of bioluminescent have been sufficiently characterized to enable transferring optimal combination of genes into Gram-negative bacteria (Winson et al., 1998). When done, resulting bioluminescent intensity of a bacterial culture is linearly proportional to bacterial cell concentration in the range from ∼10^4^ to 10^8^ ml^−1^, in comparison to OD measurements that are usually within ∼10^7^–10^8^ ml^−1^ cell concentrations (Jepson, 2014; Stevenson et al., 2016). The high accuracy of bacterial cell density measurements achieved with the bioluminescence method enabled, for example, functional classification of antimicrobial drugs according to their pairwise interactions (Yeh et al., 2006).

### Achieving Sufficient Oxygen Transfer Rate

We next discuss the importance of aeration for optimal aerobic growth. The problem can be summarized as the difference between the rate of oxygen consumption by microbes and the rate at which oxygen can be dissolved in the culture media [oxygen transfer rate (OTR)]. Similar to commercial bioreactors, to achieve fully aerobic growth, the DIY cultivation solutions need to ensure oxygen is dissolved faster than microbial cells consume it, which often means achieving a sufficiently large surface area of the culture media (Klöckner and Büchs, 2012). While OTR can be estimated (Hermann et al., 2003; Klöckner and Büchs, 2012) and several solutions to ensuring it is sufficiently high exist (Betts and Baganz, 2006), these most commonly rely on shaking rather than stirring (Klöckner and Büchs, 2012). DIY cultivation devices still predominantly stir, simply because it is technically easier and more cost-effective. Several solutions that do not require shaking could be attractive for DIY cultivation technology, for example, the introduction of baffles to the culture vessel (Gupta and Rao, 2003), or addition of immiscible oxygenated oils (Sklodowska and Jakiela, 2017). Irrespective of the solution, the specific DIY bioreactor should be calibrated to ensure fully aerobic growth in a chosen media by comparing the growth rates achieved in the bioreactor with those in fully oxygenated shaken flasks.

### Measuring Media pH and Dissolved Oxygen

Finally, a typical commercial bioreactor comes with the option of measuring pH and oxygen, where the pH value is usually monitored with an electrochemical probe [a glass or a combination electrode (Bates, 1954; Convinton et al., 1985)]. An alternative that can be integrated into DIY culturing solutions is to use fluorescent pH and oxygen indicators deposited at the bottom of the culture vessel (Wolfbeis, 1997; John et al., 2003a,b). For example, pH and dissolved oxygen level can be measured in a microbioreactor using commercially available (Presens GmbH) and platinum(II) octaethylporphine-ketone sensor spots (Papkovsky et al., 1995; Lee et al., 2011; Perez-Pinera et al., 2016). Platinum(II) and palladium(II) complexes of the porphyrin ketones exhibit strong phosphorescence that quenches in the presence of oxygen, and the spots can be embedded in polystyrene and immobilized on glass disks (Perez-Pinera et al., 2016).

## Examples

### Assessing Genetic Circuits

Evaluating the performance of genetic circuits in microplate readers is a common practice, as currently available readers come with OD, fluorescence, and luminescence measurement capabilities, and as methods that deal with auto-fluorescence from the plate and the media, as well as from the cells, have been developed (Boyer et al., 2010; Lichten et al., 2014; Mihalcescu et al., 2015). In combination with robotic liquid handling, throughput reached is sufficiently high to enable measuring of promoter activity on a genomic scale of an entire fluorescent reporter strain library (Zaslaver et al., 2006, 2009). However, commercially available plate readers with (customized) robotic liquid handling capabilities are expensive, and DIY solutions offer low cost and flexible alternative. For example, (Takahashi et al., 2014) used a 3D-printed holder of the culture vessel and a syringe pump from ABS plastic material to design a low cost “turbidostat” (**Figure 1a**). The device has in-line OD and fluorescence detection capability, implemented via a simple laser diode, light emitting diode (LED), and a photodetector.

### Adaptive Laboratory Evolution Experiments

**FIGURE 3:**
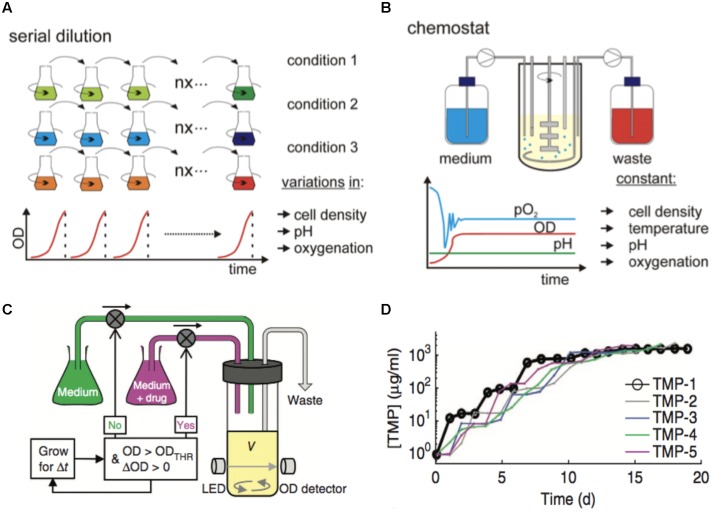
Adaptive laboratory evolution experiment. **(A)** Serial dilution passage in shaker flasks is often performed manually (Dragosits and Mattanovich, 2013). The growth parameters, such as cell density, pH, and oxygen levels can fluctuate during each growth cycle. BioMed Central. Reproduced with permission. **(B)** Chemostat operation (Dragosits and Mattanovich, 2013). The evolution experiment can also be performed with a continuous supply of media and dilution of bacterial cell density. The cell density and other environmental conditions, such as temperature, pH, and oxygen levels are kept constant. BioMed Central. Reproduced with permission. **(C)** A feedback algorithm based on the measured optical density is implemented for dynamic adjustment of the drug concentration in the morbidostat (Toprak et al., 2012). The device operates similar to the chemostat, at a given OD value it dilutes the culture by pumping extra media, except here the additional media contains higher drug concentration with each cycle. Nature Publishing Group. Reproduced with permission. **(D)** Representative results from the morbidostat (Toprak et al., 2012). The IC_50_, defined as the antibiotic inhibitor concentration at which the growth rate is 50% of the maximal growth at zero inhibitor concentration, is displayed for the drug trimethoprim over the course of 20 days. The legend shows the color codes for five parallel evolution experiments. The stepwise increase can be clearly seen. Nature Publishing Group. Reproduced with permission.

In laboratory evolution (ALE) experiments microbial cells are cultured for prolonged period of time under a chosen evolutionary pressure, by either serially diluting the culture or in a chemostat mode, **Figures 3A,B** (Lenski et al., 1991). While such experiments have been performed in laboratories for ∼30 years, recently these are being used for systems and synthetic biology and biotechnology (Buckling et al., 2009; Dragosits and Mattanovich, 2013). Common evolutionary pressures include sublethal antibiotic concentrations and growth while producing unnecessary proteins or compounds under a given expression system (Dekel and Alon, 2005).

The length of ALE experiments and the need for constant culture dilution, as well as the range of different selection pressures chosen, make them good candidates for automation and DIY design (Gresham and Dunham, 2014). For example, the “morbidostat” is a custom culturing solution that uses a feedback loop to progressively increase antibiotic drug concentration at a given measured OD, and thus keeps the drug-resistant mutant constantly challenged (Toprak et al., 2012, 2013). Using the “morbidostat” and trimethoprim as a test case antibiotic, stepwise increases of antibiotic drug resistance (up to ∼1680-fold for ∼20 days) have been observed and corresponding mutations in the drug’s target identified, **Figure 3D**. Arias Castro JC reports the design of “EvoBot,” a robot made from LEGO^®^ MINDSTORMS^®^ NXT 2.0 for automated dilution during ALE experiments (Arias-Castro, 2017). EvoBot is constructed from LEGO Bricks and controlled with RWTH Mindstorms NXT Toolbox for MATLAB (MathWorks). The culture is stirred in a 96-well plate one row at a time, where the robot inoculates every subsequent row and thus reduces the need for human intervention from once a day to approximately once a week.

### Optogenetic Intervention Bioreactors

Recently, a range of monochromatic optogenetic systems has been developed (Levskaya et al., 2005; Ohlendorf et al., 2012; Ryu and Gomelsky, 2014; Schmidl et al., 2014; Ramakrishnan and Tabor, 2016). Additionally, two-color control has been achieved (Tabor et al., 2011) and recently, three-color RGB vision has been demonstrated in *E. coli* (Fernandez-Rodriguez et al., 2017).

Light is a non-contact and versatile solution for bioprocess control and as such has been implemented in several custom culturing devices and as an add-on option to commercially available plate readers. For example, (Davidson et al., 2013) developed an array of LEDs that can be fitted to the bottom of a microplate for optogenetic control, and Tabor and colleagues constructed a Light Tube Array and Light Plate Apparatus to deliver light to individual wells on a multi-well plate (Olson et al., 2014; Gerhardt et al., 2016). The versatility that can be achieved allowed (Davidson et al., 2013) to show that a specific two-component system from cyanobacteria acts as a low pass filter when expressed in *E. coli* and (Gerhardt et al., 2016) successfully programmed specific gene expression profiles. Similarly, several groups have reported custom designed light-controlled bioreactors, using either multi-well plates (Lee et al., 2013) or larger volume vessels (Milias-Argeitis et al., 2011; Ruess et al., 2015; Wang and Yang, 2017), demonstrating switchable control of gene expression in microorganisms.

Add-on solutions to achieving optogenetic control have also been reported. For example, Moglich and colleagues modified an existing Tecan microplate reader and added optical fiber for light illumination (Richter et al., 2015; **Figure 2a**). The system has been used for *in vitro* study of *Arabidopsis thaliana* phytochrome B photoactivation, but can, in principle, be used for optogenetic control.

### Photobioreactor for Photosynthetic Microalgae

Microalgae are an important feedstock for production of useful compounds, such as biofuels and high-value chemicals (Chisti, 2007; Angermayr et al., 2015). Furthermore, their ability to capture CO_2_ while producing biomass, which in turn is a source of energy and bioproducts, has gained them increasing attention (Katiyar et al., 2017). However, microalgae are highly diverse with an estimated 200,000–1,000,000 existing species (Guiry, 2012), with each species requiring different light intensity, temperature, and carbon levels. Thus, optimizing cultivation conditions for the screening of potentially useful algae is a research challenge that can benefit from the flexibility and low cost of DIY solutions. For example, Chen and colleagues assembled optical microplates, using LEDs to supply light to individual wells and study lipid conversion efficiency of *Dunaliella tertiolecta*. Similarly, the recently developed “Photobiobox” integrates 96-well plate with LEDs for illumination, coupled with light gradient filters, and water blocks to achieve light intensity and temperature gradients (Heo et al., 2015), **Figure 2c**. The device was used to screen 12 microalgae strains from fresh water for their growth and lipid production potential. Add-on solutions have also been proposed, Morschett and colleagues modified a commercial microplate reader for 48-fold parallelized algae cultivation by incorporating a “photo module” for individual well illumination (Morschett et al., 2017).

### Anaerobic Cultivation

Anaerobic cultivation of microorganism is of increasing interest in industrial biotechnology, e.g., bacterium *Clostridium* that biochemically synthesizes solvents (Green, 2011). Most commonly, anaerobic cultivation is achieved with the use of specialized anaerobic cabinets (Leach et al., 1971). While agar plate growth can be performed in anaerobic jars (Brewer and Brown, 1938; Brewer, 1942) and gas packs, and commercially available tubes with rubber stoppers can be used for liquid culture growth (Anderson and Fung, 1983), using these without the anaerobic cabinets makes it difficult to monitor culture growth (for example, measure OD). We envision that DIY culturing technology can simplify anaerobic cultivation and enable cheap and innovative solutions. For example, recently reported *Moorella thermoacetica* that can self-photosynthesize via synthetically introduced cadium sulfur (CdS) nanoparticles, is strictly an anaerobe (Drake and Daniel, 2004; Sakimoto et al., 2016). Thus, the reported photosynthesis requires cultivation under anaerobic conditions in the presence of light. DIY solutions could be designed to fit the purpose, e.g., bioreactors can be furnished with LED light and specific gas source. Low power electronic components such as LEDs and semiconductor photodetectors can run on battery power, and transmission of data collected can be achieved with wireless communication, which leaves the possibility of designing solutions that can be placed in specialized environments as well. We, therefore, believe DIY solution for anaerobic growth can widen the extent and ease with which such microorganisms are cultured.

## Conclusion

We have surveyed some of the DIY solutions for microbial cultivation as summarized in **Table 1**. While no single solution satisfies all the requirements of different research communities, our intention was to demonstrate that DIY solutions are becoming ever more cost-effective and user-friendly and can expand the range of experiments that can be performed in a given laboratory. We would also like to draw the reader’s attention to the “Open-Labware” collections that are aiming to bring together different DIY projects beyond, but including, cultivation technologies (Baden et al., 2015). We anticipate this community to grow and offer further improved and increasingly versatile designs.

**Table 1:** Summary of DIY bioreactor and microplates.

Name	In-line detection	Volume	Organism	Note	Reference
Turbidostat	OD, fluorescence	15 ml	Yeast	3D printed holder and pump	Takahashi et al., 2014
Morbidostat	OD	12 ml	*E. coli*	Antibiotic drug resistance	Toprak et al., 2012, 2013; Liu et al., 2016
Microreactor	OD, PH, and oxygen	1 ml	*E. coli*	Point of care	Lee et al., 2011; Perez-Pinera et al., 2016
Optogenetic bioreactor	OD fluorescence	12 ml	*E. coli*	Optogenetics	Wang and Yang, 2017
Light tube array (LTA)	None (fluorescence with offline flow cytometry)	1 ml	*E. coli*	Optogenetics for bacteria and mammalian cell entrapment of cyanobacteria	Olson et al., 2014; Gerhardt et al., 2016
Cell growth quantifier (CGQ)	OD	50 ml	Yeast	Back scattering and high speed data acquisition	Bruder et al., 2016
Microplate with robotic assay	OD, fluorescence	∼100 μl	*E. coli*	Gene expression profiling	Zaslaver et al., 2006, 2009; Huber et al., 2009
Optical Microplate	None (fluorescence with offline flow cytometry)	100 ml	*E. coli Dunaliella tertiolecta*	Photosynthesis optogenetics	Chen et al., 2012; Davidson et al., 2013; Lee et al., 2013
PhotobioBox	OD	200 ml	Microalgae	Light intensity and temperature gradient between the wells	Heo et al., 2015

*Several design features of each solution are listed for comparisons, e.g., working volume, organism the cultivation is designed for an in-line detection capabilities. Under the “Note” column we have listed the main intended application or any special features of the solution.*

## Author Contributions

Y-TY conceived the original idea. TP and Y-TY wrote the manuscript.

## Conflict of Interest Statement

The authors declare that the research was conducted in the absence of any commercial or financial relationships that could be construed as a potential conflict of interest. The reviewer PS and handling Editor declared their shared affiliation.
